# Fatigue as a complicating factor in the recovery of breast cancer survivors treated at an oncology clinic in South West Nigeria: a case–control study

**DOI:** 10.3332/ecancer.2022.1420

**Published:** 2022-07-04

**Authors:** Sharif Adeniyi Folorunso, Atara Ntekim, Abbas Adesina Abdus-salam, Adeniyi Olabumuyi, Aminat Omolara Folorunso, Festus Igbinoba, Adeniyi Abidemi Adenipekun

**Affiliations:** 1 Department of Radiology, Obafemi Awolowo University Teaching Hospital, Ile Ife, 220282, Nigeria; 2 Department of Radiation Oncology, University College Hospital/University of Ibadan, 200212, Nigeria; 3 Department of Radiation Oncology, University College Hospital Ibadan, 200212, Nigeria; 4 Department of Chemical Pathology Obafemi Awolowo University Teaching Hospital, Ile Ife, 220282, Nigeria; 5 National Hospital Abuja, 900211, Nigeria

**Keywords:** breast, cancer, survivors, fatigue

## Abstract

**Purpose:**

Recovering cancer survivors hope to return to their premorbid lifestyle after treatment and be free from the disease. They are, however, faced with some psychosocial issues, including fatigue, which could negatively impact their quality of life. With increasing cancer awareness and improvement in treatment, it is expected that the number of cancer survivors will increase in Nigeria. Little has, however, been done with regard to survivorship care in the country. It is important to explore fatigue in this group of patients with a view to find ways of reducing it to the barest minimum.

**Aim:**

To assess the level of fatigue in breast cancer survivors on follow-up visit at a radiation oncology clinic and compare it with age and sex-matched apparently healthy controls.

**Materials and Methods:**

Fatigue levels were obtained using the Fatigue Symptom Inventory (FSI). Kruskal–Wallis H test was used to compare the FSI scores in cases and controls. Chi-squared test was used for comparison of proportions. Level of significance was set at 5%.

**Results:**

Seventy cancer survivors (cases) and 70 apparently healthy age (±1)-matched controls were recruited. The prevalence of fatigue was higher among cases than controls (24.3% versus 10%; *p* = 0.025). Breast cancer survivors reported significantly worse fatigue on the day they were most fatigued (*p* = 0.017), least fatigued (0.047) and fatigued on average (*p* = 0.006) compared to controls. Fatigue also significantly interferes with the ability to concentrate (*p* = 0.040) and relate with people (*p* = 0.002) more in cases compared to controls. While fatigue was more common in the morning and afternoon in breast cancer survivors, fatigue either occurred more in the evening or followed no daily pattern in the controls.

**Conclusion:**

Breast cancer survivors reported worse fatigue, suggesting the need to include fatigue screening as part of post-treatment follow-up. There is also a need to investigate the factors responsible for this and explore ways of reducing or eliminating it.

## Introduction

Breast cancer diagnosis and treatment come with a huge burden on patients. Recovering cancer survivors hope to return to their premorbid lifestyle after treatment and be free from the disease. They are, however, faced with some psycho-social issues, including fatigue, which could be distressing, negatively impact their quality of life and reduce their productivity [1]. Some patients reported that addressing behavioural symptoms, such as fatigue, is as important as treating the cancer itself [5].

Fatigue is the most frequently reported symptom of cancer patients [2, 3]. It is reported in about 90% of breast cancer patients receiving radiation and chemotherapy, regardless of whether the cancer is invasive or non-invasive, with residual fatigue often persisting months after the completion of treatments [4–6]. Although healthy individuals also experience fatigue, fatigue related to cancer is persistent, disproportionate to recent activity, interferes with normal activity and is not relieved by rest [1, 5]. Studies have shown that the intensity and duration of fatigue experienced by breast cancer patients and survivors are significantly greater than healthy controls and could cause greater impairment in the quality of life [1, 4, 5]. Fatigue can also be a predictor of recurrence-free survival and overall survival in breast cancer [7].

Most studies on cancer patients in developing countries, like Nigeria, focus on prevention, treatment and palliative care, while survivorship receives less attention. This could be due to poor survival that results from late presentation, aggressive tumour biology and a dearth of facilities for cancer treatment [8]. With increasing cancer awareness, and advances in diagnosis and treatment, it is expected that the number of breast cancer survivors in Nigeria will increase significantly in the years to come [9]. Little has, however, been done as regards survivorship care in the country. To the best of our knowledge, no study has assessed the severity of fatigue in breast cancer survivors in Nigeria. It is, therefore, important to explore fatigue in this group of patients in order to find ways to reduce it to the barest minimum. The aim of this study was to assess the level of fatigue in breast cancer survivors on follow-up visits and compare the findings with age and sex-matched apparently healthy controls.

## Methodology

### Study design

This study was a case–control study.

### Setting

The study was conducted at the University College Hospital Ibadan, South West Nigeria, between 06 September 2019 and 05 September 2020. Ethical approval for the study was obtained from the institution’s ethics committee. Informed consent was obtained from the participants before enrollment.

### Participants

The inclusion criteria for cases included patients who completed curative treatment (surgery, chemotherapy and radiotherapy) for histologically confirmed breast cancer, not less than 3 months previously and were on follow-up (at least second visit post-treatment) with no clinical evidence of the disease. Controls were age and sex-matched apparently healthy patients attending the general outpatient department (GOPD). Patients with other forms of cancer and patients with fever were excluded. Patients with a known untreated or unstable medical condition, such as poorly controlled diabetes mellitus, high blood pressure, renal disease, mental illness and HIV infection and other chronic infections, were also excluded.

### Variables

The magnitude of fatigue was assessed in cases and controls.

### Data measurement

The Fatigue Symptom Inventory (FSI) was used to quantify fatigue in the participants. FSI is available in the original article published by Hann *et al* [11]. FSI has been validated in both men and women, across a wide age range, including the elderly, and in a variety of cancer diagnoses, including patients in active treatment and survivors [12]. Based on a systematic review, Cronbach’s alpha coefficients of the subscale of FSI ranged from 0.84 to 0.96 [12]. The FSI has, moreover, demonstrated test–retest reliability, construct validity, divergent validity, convergent validity, and discriminant validity [11, 13]. The scale also possesses reliability and validity with healthy controls [11]. It has been used to assess fatigue among Caucasians, African Americans, Chinese, Indians and Arabs receiving cancer treatment [4, 14, 15]. The scale was used in a similar study by Kumar *et al* [4] while studying thyroid function test in the aetiology of fatigue in breast cancer survivors. However, studies that used this scale among Nigerians is scarce.

A score of 3 or greater on the average fatigue severity item or a mean score of 3 or greater on those items assessing fatigue severity in the past week is the recommended cut-off for discriminating cases of clinically meaningful fatigue from non-cases [11, 12].

### Study size

*N* = (*Z*_α_+*Z*_β_)^2^ × 2 × (SD^2^) / (*µ*_1−_*µ*_2_)^2^

where:

*N* = Desired sample size;

*Z*_α_ = Two-sided percentage point of the normal distribution corresponding to level of significance = 1.96;

*Z*_β_ = Percentage point of the normal distribution corresponding to a power of 80% = 0.84;

SD = Standard deviation of the FSI of a relevant previous study. The SD of the mean FSI in a previous study [10] was 1.84;

*µ*_1−_*µ*_2_ = Mean difference of the FSI the study aims to detect between cases and controls where half of the standard deviation = 0.92;

Therefore, *N* = (1.96+0.84)^2^ × 2 × (1.84^2^) / (0.92)^2^ = 62.72 (approximately 63).

Adjusting for a non-response of 10%, 63 will be divided by 0.9 which gives 70, so the estimated sample size is 140 (70 cases and 70 controls).

One hundred and forty patients were recruited. Seventy were cancer survivors (cases) and 70 were apparently healthy age (±1) and sex-matched controls.

### Statistical methods

Data were collected by trained research assistants, and were collated, computed, coded and subjected to statistical analysis using the IBM Statistical Package for the Social Sciences v21. The Kruskal–Wallis H test was used to compare FSI scores in cases and controls because data were not normally distributed. Chi-squared test was used for comparison of proportions. Level of significance was set at 5%.

## Results

Seventy breast cancer survivors (cases) and 70 apparently healthy age (±1)-matched controls were recruited. The mean age of the cases was 51.26 ± 8.62 years, while the mean age of the controls was 51.17 ± 8.58 years. Most of the patients (53, 75.7%) were aged between 40 and 59 years in both the cases and the controls. They were all female.

**Figure 1.**
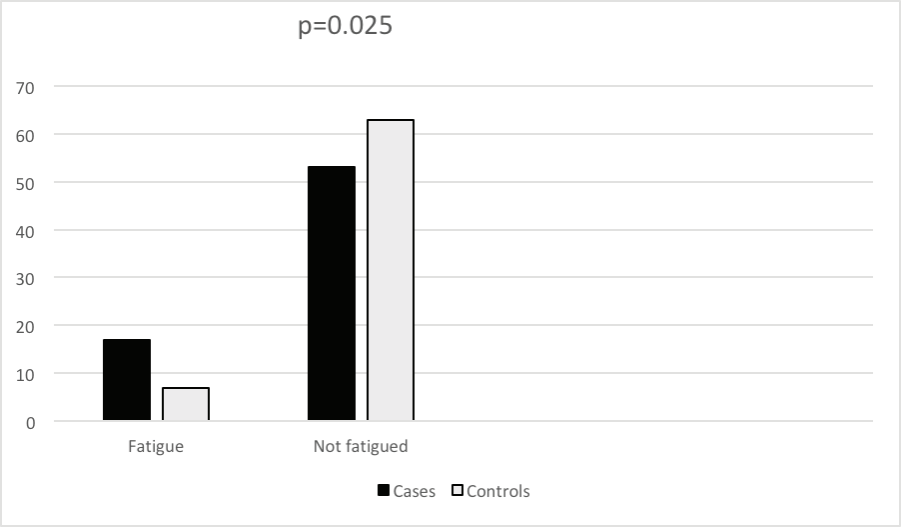
Frequency of fatigue in breast cancer cases and controls.

The Fatigue Symptom Inventory was used to assess fatigue in both cases and controls. Seventeen (24.3%) cases and 7 (10%) controls reported clinically meaningful fatigue. There was a significant difference between the two arms (*p* = 0.025) (see Figure 1).

In term of the severity of fatigue, breast cancer survivors reported significantly worse fatigue on the day they were most fatigued (*p* = 0.017), least fatigued (*p* = 0.047) and fatigued on average (*p* = 0.006). Fatigue also significantly interfered with the ability to concentrate (*p* = 0.040) and relate with people (*p* = 0.002) more in cases compared with controls. The other parameters in the fatigue symptom inventory, however, show no statistically significant difference between cases and controls (see Table 1).

**Figure 2.**
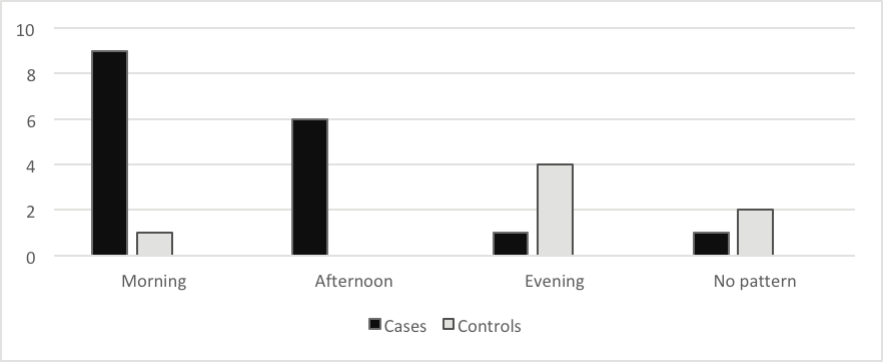
Time of the day when fatigue is worse in cases and controls.

Figure 2 shows the daily pattern of fatigue. While fatigue was more common in the morning and afternoon in breast cancer survivors, fatigue either occurred more in the evening or followed no daily pattern in the controls (*p* = 0.001).

## Discussion

It was observed that the majority of breast cancer survivors were between 40 and 59 years of age, with a mean of 52.26 years. This differs from the findings in the United States where more than 75% of the breast cancer survivors were 60 years and above [16]. This may not be unconnected with the fact that breast cancer is diagnosed at a younger age in Nigeria when compared to developed countries [17–19]. While the mean age of diagnosis of breast cancer is 47.5 years in Nigeria [18], the mean age at diagnosis is 61 years in the United States [20]. This shows that the majority of breast cancer survivors in this environment are in the age group considered to be the most productive working force in the society [21]. Hence, support systems should be in place for working class cancer survivors to reduce the socio-economic impact of cancer diagnosis [20, 21]. The unique medical and psychosocial needs (like fatigue) of cancer survivors require proactive assessment and management [20].

All the 70 breast cancer survivors recruited were female. This is not unexpected as male breast cancer remains a rare entity with a prevalence rate of less than 1% [22].

The prevalence of fatigue in cases was significantly higher when compared with the controls. The severity of fatigue was also worse in breast cancer survivors. Previous studies have shown that the frequency, intensity and duration of fatigue experienced by breast cancer patients and survivors is significantly greater than healthy controls and could cause greater impairment in the quality of life [1, 4, 5, 23]. To the best of our knowledge, no study in Nigeria has examined the frequency and severity of fatigue in breast cancer survivors. Similar to the findings of other studies carried out outside the country, it can be deduced from this study that fatigue is also a major problem in breast cancer survivors in Nigeria. The frequency and severity of fatigue in breast cancer survivors suggests the need to include fatigue screening as part of the assessment when patients present for follow-up [3, 4].

Studies have linked fatigue in breast cancer survivors to the sequel of cancer treatment (especially systemic therapy), although the direct effect of such treatments on fatigue has not been agreed upon [24]. Fatigue in breast cancer survivors could also result from the disease itself as significantly worse fatigue has been reported in survivors of some other chronic diseases [25]. For example, a study reported post-stroke fatigue to be as high as 82% [26]. Psychological factors, such as the fear of recurrence, can also worsen fatigue in breast cancer survivors [27]. Although fatigue reported in this study results from the above-mentioned factors could not be ascertained, we hope to examine the determinants of fatigue in our future studies.

Fatigue was reported more in the morning and afternoon in the breast cancer survivors but more in the evening or followed no daily pattern in the controls. This is similar to the findings of previous studies [28, 29]. The implication of this is that breast cancer patients having fatigue in the morning could have difficulties with the ability to work. Morning and afternoon are periods that most people are at work; hence, having fatigue during these periods may reduce the productivity of cancer survivors and enhance their continued dependence on caregivers despite being free from disease. This shows that paying attention to this symptom will not only solve the plight of the survivors but also their caregivers and even the economy of the nation.

Although the reason for this variation has not been fully elucidated, it has been suggested that while fatigue in the evening often results from normal physiologic response to daily activity, morning fatigue could be pathologic, resulting from poor sleep or anxiety [28, 30]. Checking behaviour and fear of cancer recurrence are top ongoing concerns in breast cancer survivors that can induce anxiety and poor sleep [31]. Whether these were the reasons for the morning fatigue was not addressed in this study, suggesting the need to further explore morning fatigue in breast cancer survivors.

## Conclusion

Breast cancer survivors reported worse fatigue compared with the control group. Fatigue occurred commonly in the morning and afternoon in cases but in the evening or followed no pattern among the controls. This suggests the need to include fatigue screening as part of the assessment when patients present for follow-up. There is also a need to investigate the factors responsible for this and explore ways of reducing or eliminating it. Further studies are suggested in this regard.

## Limitation

Being a case–control study, patients were questioned about fatigue they experienced retrospectively within the last week; this may introduce some recall bias into participants’ responses.

The study compared the severity of fatigue in breast cancer survivors and apparently healthy controls; however, the determinants of fatigue were not explored. Future studies are recommended with the hope to assess the determinants of fatigue in breast cancer survivors in Nigeria.

## Conflicts of interest

None.

## Funding

None.

## Figures and Tables

**Table 1.** Summary of the severity of fatigue symptoms in breast cancer cases and controls.

	Fatigue Symptom Inventory	Case scores median (IQR^b^)	Control scores median (IQR^b^)	*p* value
**1**	Most fatigued during the past week	1 (0,5)	0 (0,3)	0.017^a^
**2**	Least fatigued during the past week	0 (0,1)	0 (0,0)	0.047^a^
**3**	Fatigue on the average during the past week	1 (1,2)	0 (0,0)	0.006^a^
**4**	Fatigue right now	0 (0,1)	0 (0,0)	0.364
**5**	Fatigue interfered with your general level of activity	0 (0,0)	0 (0,1)	0.265
**6**	Fatigue interfered with your ability to bathe and dress yourself	0 (0,0)	0 (0,1)	0.332
**7**	Interfered with your normal work activity	0 (0,1)	0 (0,0)	0.446
**8**	Fatigue interfered with your ability to concentrate	0 (0,1)	0 (0,0)	0.040^a^
**9**	Fatigue interfered with your relations with other people	0 (0,1)	0 (0,0)	0.002^a^
**10**	Fatigue interfered with your enjoyment of life	0 (0,0)	0 (0,0)	0.195
**11**	Fatigue interfered with your mood	0 (0,0)	0 (0,0)	0.489
**12**	How many days, in the past week, you felt fatigued	0 (0,2)	0 (0,1)	0.501
**13**	How much of the day, on average?	0 (0,2)	0 (0,1)	0.281
	Disruption Index (Total sum of items 5-11)	0 (0,5)	0 (0,4)	0.430

Kruskal–Wallis test was used

a Significant

b Interquartile range
